# Supplementary material for: AffyMAPSDetector: a software tool to characterize Affymetrix GeneChip™ expression arrays with respect to SNPs
Source: BMC Bioinformatics. 2007 Jul 30;8:276. doi: 10.1186/1471-2105-8-276 (PMC1959249; doi:10.1186/1471-2105-8-276)
Supplement: Additional file 13 — AffyMAPSDetector v1 source code. AffyMAPSDetector source code. [file 1471-2105-8-276-S13.zip › AffyMAPSDetector_v1_src/doc/index-files/index-19.html]

V-Index


|  |  |  |  |
| --- | --- | --- | --- |
| **Overview** \| Package \| Class \| Use \| **Tree** \| **Deprecated** \| **Index** \| **Help** | | |  |
| **PREV LETTER**   **NEXT LETTER** | **FRAMES**    **NO FRAMES**     **All Classes** |  |  |


A B C D E F G H I J L M N O P R S T V W X Y 

---


## **V**

**VIEW\_FILTERED\_PROBES** - Static variable in class affychip.snpdetector.Constant: **VIEW\_REMOVED\_RPOBES** - Static variable in class affychip.snpdetector.Constant: **validateFinish(List)** - Method in class affychip.snpdetector.ui.StepOnePanel: Called to validate the panel before finishing the wizard. **validateFinish(List)** - Method in class affychip.snpdetector.ui.StepThreePanel: **validateFinish(List)** - Method in class affychip.snpdetector.ui.StepTwoPanel: **validateFinish(List)** - Method in class jwf.NullWizardPanel: Called to validate the panel before finishing the wizard. **validateFinish(List)** - Method in class jwf.WizardPanel: Called to validate the panel before finishing the wizard. **validateNext(List)** - Method in class affychip.snpdetector.ui.StepOnePanel: Called to validate the panel before moving to next panel. **validateNext(List)** - Method in class affychip.snpdetector.ui.StepThreePanel: **validateNext(List)** - Method in class affychip.snpdetector.ui.StepTwoPanel: **validateNext(List)** - Method in class jwf.NullWizardPanel: Called to validate the panel before moving to next panel. **validateNext(List)** - Method in class jwf.WizardPanel: Called to validate the panel before moving to next panel.

---


|  |  |  |  |
| --- | --- | --- | --- |
| **Overview** \| Package \| Class \| Use \| **Tree** \| **Deprecated** \| **Index** \| **Help** | | |  |
| **PREV LETTER**   **NEXT LETTER** | **FRAMES**    **NO FRAMES**     **All Classes** |  |  |


A B C D E F G H I J L M N O P R S T V W X Y 

---
